# The biomechanics of splitting hairs

**DOI:** 10.1098/rsfs.2023.0063

**Published:** 2024-06-07

**Authors:** David Taylor, Ellen Barton, Isobel Duffy, Ramona Enea-Casse, Guillaume Marty, Robert Teeling, Roberto Santoprete

**Affiliations:** ^1^ Trinity Centre for Biomedical Engineering, Trinity College Dublin, University of Dublin, Dublin, Ireland; ^2^ L’Oréal Centre Charles Zviak, Saint-Ouen 93400, France; ^3^ L’Oréal Aulnay, Aulnay-sous-Bois 93601, France

**Keywords:** hair, break, split, strength, toughness, fatigue

## Abstract

Splitting of hair, creating ‘split ends’, is a very common problem which has been extensively documented. However, the mechanics underlying the splitting phenomenon are poorly understood. This is partly owing to the lack of a test in which splitting can be generated and quantified under laboratory conditions. We developed three new tests, known as ‘loop tensile’, ‘moving loop’ and ‘moving loop fatigue’, aiming to simulate the mechanical environment of tangles of hair strands during combing. We tested straight strands of human hair, comparing low-quality hair (from a subject who experienced split ends) with hair from a control (non-splitting) subject. Significant differences were found, especially in the moving loop fatigue test where the low-quality hair failed in fewer cycles. Splitting occurred in both types of hair, but with the crucial difference that in the low-quality hair, splits originated inside the hair strand and propagated longitudinally over considerable distances, while in the control hair, splits originated at the strand surface and remained short. Bleaching of the control hair changed its behaviour, making it similar to that of the low-quality hair. Some simple calculations emphasized the role of longitudinal shear stress and shear stress intensity in generating microcracks which could then propagate within the moving loop, paving the way for a future theoretical model of the splitting mechanism.

## 1. Introduction

Damage and fracture of human hair is a very common phenomenon which occurs during everyday activities such as combing and brushing. In particular, many people complain of the problem of ‘split ends’ in which the end of the hair strand divides into two or more sub-strands. The problem may occur naturally but is often linked to cosmetic treatments such as bleaching, straightening and blow-drying. Many papers have been published on hair damage (see below) but there is considerable controversy about the causes of damage and fracture and of the appropriate methods for its mitigation. The problem is made more complex by the particular mechanical properties of hair, which include nonlinear stress/strain behaviour and anisotropy. Splitting is essentially a fracture mechanics problem, involving the initiation, and subsequent propagation, of a crack oriented longitudinally to the hair strand. However, to date, very few fracture mechanics studies have been conducted.

The aims of the present work were:

1. To review the literature on the mechanical properties and fracture of human hair, and also of other keratin-based natural materials.
2. To review existing theories on the mechanisms underlying hair breakage and splitting.
3. To devise experiments to generate splitting under laboratory conditions in order to quantify the effects of hair type and treatment on the tendency to form splits.

### 1.1. Structure of hair

**Figure 1.**
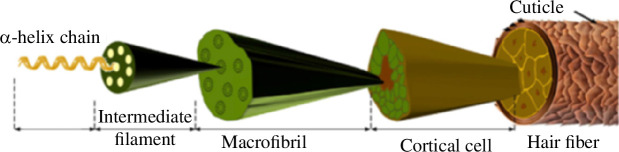
Structure of human hair, from [1].

Figure 1 shows the structure of a hair strand, which consists of an outer layer of cuticle surrounding the cortex which takes up most of the volume. The diameter is typically 40–150 μm; the cross-section varies from circular to elliptical, with ellipse ratios typically 1.0–1.4 [1]

The cortex is made up of typically 90% keratin, much of it in the crystalline, α form, arranged as fibres of long aspect ratio, lying parallel to the hair axis. Matrix material (consisting largely of amorphous proteins and lipids) lies between the fibres. In detail, the structure is complex, involving several hierarchical levels, with fibres bundled together and surrounded by matrix, at scales varying from nanometres to micrometres. Two important scales are (i) the intermediate filaments (IFs), which are assemblies of keratin molecules which form a microfibril of diameter about 7 nm, and (ii) the bundles of fibres known as cells, of diameter about 5 μm, connected together by the cell membrane complex (CMC), where most fractures seem to originate [2].

The cuticle consists of a series of sheets, arranged in an overlapping fashion resembling tiles on a roof. There are typically 6–8 layers of tiles, giving an overall thickness of 3–4 μm, made from keratin in the β form, connected by matrix material to each other and to the cortex. From a mechanical point of view, it is the cortex which provides the great majority of the stiffness and strength. The role of the cuticle is largely to protect the cortex from physical damage and chemical degradation.

### 1.2. Mechanical properties of hair and other keratin-based materials

**Figure 2.**
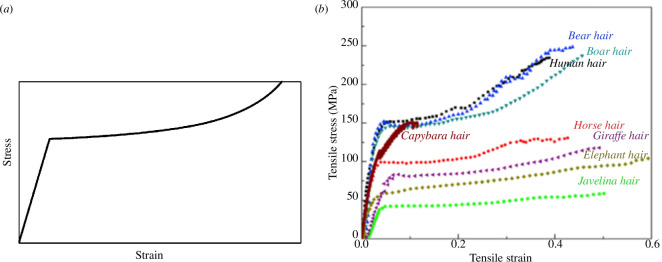
(*a*) Simplified stress/strain curve for hair. (*b*) Examples of results from human and animal hair [1].

The most common test used to measure the mechanical properties of hair is the tensile test, in which a single hair (known as a strand or fibre) is clamped at each end and stretched. Figure 2 shows a typical stress/strain curve in schematic form, and experimental results for hair from humans and other animals [1]. Initial loading shows linear, elastic behaviour with some curvature at increasing stress which is likely owing to viscoelasticity. After a clear yield point, the behaviour becomes highly nonlinear, characterized by an upturning stress/strain curve which continues to exceptionally high strains, up to 50%. This is owing to the α-keratin of the cortex gradually unwinding and undergoing a phase change to β-keratin, with an accompanying increase in volume.

Results in the literature vary, and often show considerable scatter even within samples from the same source, especially with regard to the stress and strain at failure [1–5]. Typically, Young’s modulus lies in the range 2–4 GPa, yield strength 50–200 MPa, ultimate strength at failure 100–400 MPa and failure strain 20–50%. Humidity is a significant factor: the values given here apply at normal, ambient humidity levels (30–65% relative humidity). Viscoelastic behaviour has been demonstrated by carrying out tensile tests at different strain rates [6] and using other techniques such as dynamic mechanical analysis [7]. Elastic modulus and strength increase significantly with the loading rate.

In addition to tensile tests, a few papers describe other types of mechanical testing. Shear tests have been conducted by subjecting the hair strand to torsion [8,9]. One group has carried out impact tests in which one hair strand was dropped with an attached weight, so that it struck a stationary strand or the tooth of a comb [10]. Cyclic tests have been carried out to investigate fatigue behaviour, though to date, only a small number of such investigations have been conducted, showing failure after 20 cycles at high applied torsional strains [11], after thousands of cycles in torsion [12] and hundreds of thousands of cycles in tension [13]. These demonstrate that fatigue can occur at loadings much less than that required for instantaneous failure, but a complete picture of fatigue failure in this material has not yet been established.

It is clear from examination of damaged and broken hair that the principal mechanism of failure is cracking. Small cracks form and grow in different directions through the hair strand. This suggests that fracture toughness and fatigue crack propagation may be important parameters, but to date, there have been very few attempts to measure the material’s fracture toughness, and no work on fatigue crack propagation. Kamath and Weigmann examined fracture surfaces after tensile testing: they noticed a relatively smooth region covering about one-third of the cross-section which they hypothesized to be slow crack growth occurring before the crack reached a critical length for rapid, brittle fracture [14]. In this way, they estimated a crack propagation energy *G*_c_ for transverse crack growth of 1.7 kJ m^−2^.

A major limitation of this previous work is that it only provides information from tests in which hair is loaded in the longitudinal direction. We can expect hair to be highly anisotropic, given that it consists largely of oriented fibres, but it is difficult to devise tests in which hair is subjected to transverse loading, which will of course be crucial for an understanding of splitting. In the absence of such data, it is useful to look at results from other natural materials made from keratin, for which there is a large body of work. What emerges is that the degree of anisotropy is a strong function of the size scale on which the measurement it made. Tests at the very small scale of the IFs showed that they had Young’s modulus which was 100 times greater than the surrounding matrix material [15], implying a degree of anisotropy in stiffness of about 100, since in tests conducted perpendicular to the fibre direction deformation will occur mainly in the matrix. At a somewhat larger scale, stiffness anisotropy was found to be 20 for horse hair [16] and 10 for small bundles of keratin fibres taken from animal hooves [15]. Moving to a more macroscopic scale, porcupine quills were found have anisotropy of 3.4 in stiffness [17]. Tests on human fingernails found a shear modulus *G* which was 10 times lower than the Young’s modulus *E* measured in the stiff direction [18]. Interestingly, these various measurements showed similar values for the longitudinal Young’s modulus, in the range 2–4 GPa, independent of source and scale.

Strength anisotropy has not been as extensively measured as stiffness anisotropy. Taking experience from engineering fibre composites, one would expect the anisotropy in strength to be similar to that in stiffness, and some support for this comes from the work of Chou *et al.* on porcupine quills mentioned above. They compared values of Young’s modulus, yield strength and ultimate tensile strength in the longitudinal and transverse directions. The measured anisotropy in yield strength and ultimate tensile strength was 2.7 and 2.5, respectively, comparable to the stiffness anisotropy which was 3.4. Larger structures tend to show less anisotropy: for example, bovine horn keratin had an anisotropy factor of 2 in both Young’s modulus and strength [19].

Turning to measures of fracture toughness, a relevant keratin-based material is the human fingernail. It consists of three layers but the principal layer is made up of highly oriented α-keratin. It is for this reason that nails crack much more easily in the direction perpendicular to the axis of the finger. Farran *et al.* conducted toughness measurements by recording the energy needed to cut nail samples with a scissors [18]. They found crack propagation energies (*G*_c_) of 4 J m^−2^ and 1 kJ m^−2^ for the high and low toughness directions, respectively, giving an anisotropy factor of 4. Toughness tends to be larger and less anisotropic in structures of greater size, such as hooves and horns [20], owing to their more complex structures consisting of layers in which keratin fibres run in different directions.

Fibrous materials tend to be weaker in compression than tension owing to the tendency of fibres to buckle. For example, wood is 2–3 times stronger in tension than in compression when tested parallel to its grain [21]. The same might be expected of hair, but this has not been tested. In fact, there are few records of compression tests conducted to failure on any keratin-based material. Exceptionally, Yang and McKittrick tested samples of porcupine quill, removing the inner soft material and loading the cortex in axial compression [22]. They measured a Young’s modulus of 2.6 GPa, similar to tensile values. Their samples were loaded up to 135 MPa and at that point failed by buckling of the tubular structure. So, it can be assumed that the true compressive strength of the keratin was greater than this figure, and thus at least similar to the yield strength of hair keratin in tension.

### 1.3. Damage and failure modes

**Figure 3.**
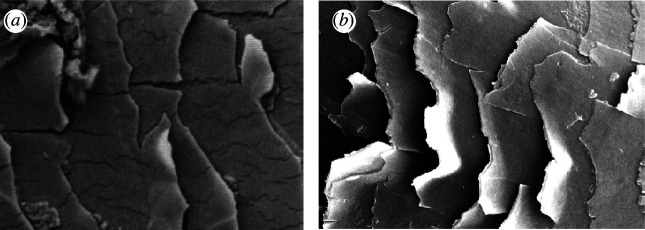
Examples of cuticle surface damage. (*a*) Longitudinal cracking [23]; (*b*) lifting of cuticle tiles [24].

Many workers have observed and described damaged and fractured hair using scanning electron microscopy. Early signs of damage include small surface cracks which are usually oriented longitudinally (figure 3*a*) [23] and lifting of cuticle tiles (figure 3*b*) [24]. Cuticle has a much lower strain to failure than cortex and so will tend to separate from the cortex during tensile loading, long before the cortex itself fails.

Hair fracture occurs by crack propagation, which can be transverse, longitudinal or a mixture of the two, leading to different appearances of the resulting fracture surface. A useful classification of these fracture modes [14] is as follows:

**Figure 4.**
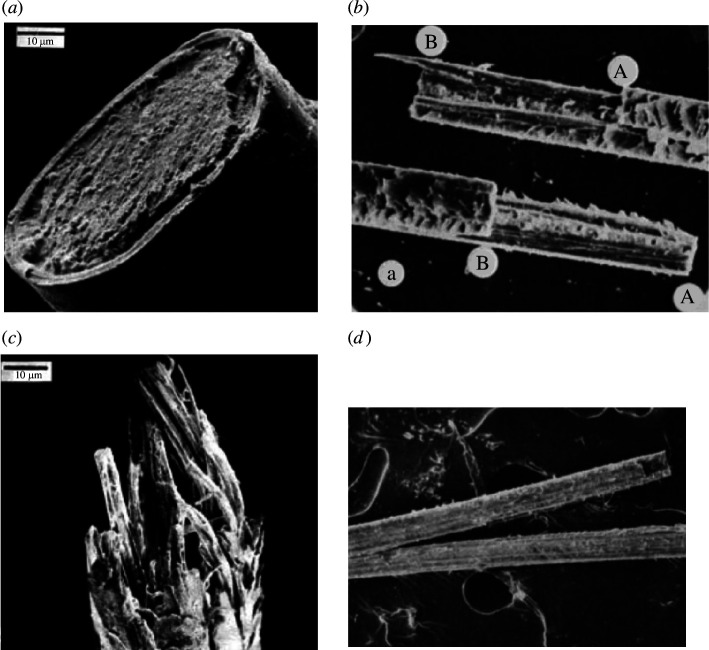
Examples of the four modes of hair fracture: (*a*) flat [25]; (*b*) stepped [14]; (*c*) fibrous [25]; (*d*) splitting [14].

- *Flat fractures* caused by transverse crack propagation, creating a fracture plane which is perpendicular to the hair axis (figure 4*a*).
- *Stepped fractures* involving both transverse and longitudinal cracking (figure 4*b*). The length of the longitudinal (i.e. splitting) part of the fracture is typically less than 1 mm.
- *Fibrous fractures*, which have the appearance of a paintbrush (figure 4*c*). Though macroscopically transverse, they are characterized by multiple short longitudinal fractures.
- *Splitting* is caused by extended propagation of a single crack in the longitudinal direction (figure 4*d*).

Normal hair has been found to fail by flat fracture near the root and with one of the other three modes (stepped, fibrous or split) near the tip [25]. The flat fracture mode is regarded as being typical of good-quality hair, while the other modes appear in hair which is degraded by environmental attack or by treatments such as bleaching or permanent waving. There is evidently a competition between different fracture modes.

### 1.4. The effect of treatments and environmental factors

Much has been written about the effects of everyday actions (e.g. brushing, drying [24] and sunlight [26]) and cosmetic treatments (e.g. bleaching [26], curling [27] and dying [28]) as well as the effects of products intended to reduce or repair damage [29]. Physical abrasion can damage and even completely remove cuticle, as mentioned above, and combing and brushing are believed to be major causes of fracture, as will be described in more detail below. Chemical effects cause weakening of the CMC and its adhesion to the keratin fibres in both cortex and cuticle. Two principal bonding types which are affected are hydrogen bonds and cysteine bonds. Wetting of hair weakens hydrogen bonds, causing it to be more flexible and more prone to physical damage, but this weakening is reversible when the hair dries. Breakage of the stronger cysteine bonds can occur owing to chemical treatments such as bleaching, permanent wave treatments and treatments to straighten curly hair [27]. This causes a loss of strength which is permanent unless these bonds are restored by subsequent chemical treatment.

The effect of these factors has been quantified using mechanical testing, with differing results. Yuen *et al.* recorded reductions of about 20% in the tensile failure strength as a result of treatments (perming or bleaching) and exposure to artificial sunlight [26]. Beyak *et al.* reported larger effects, exceeding 50% reduction in the stress for a strain of 15% (essentially the yield strength) following bleaching and/or UV irradiation [30]. Harper and Kamath measured a 35% reduction in torsional shear modulus [8] owing to bleaching.

### 1.5. Theories of hair fracture

In considering the biomechanics of hair fracture, the first problem is to identify sources of mechanical loading which are capable of generating enough stress in the hair strand to cause failure. A simple calculation can show that pulling the hair in axial tension is very unlikely to cause it to fail. The force needed is high (about 1 N), so high that the hair will be pulled out by the root before tensile failure occurs [31]. Brushing and combing will create shear stress owing to friction, but this will be very small in an individual hair which is free to move.

**Figure 5.**
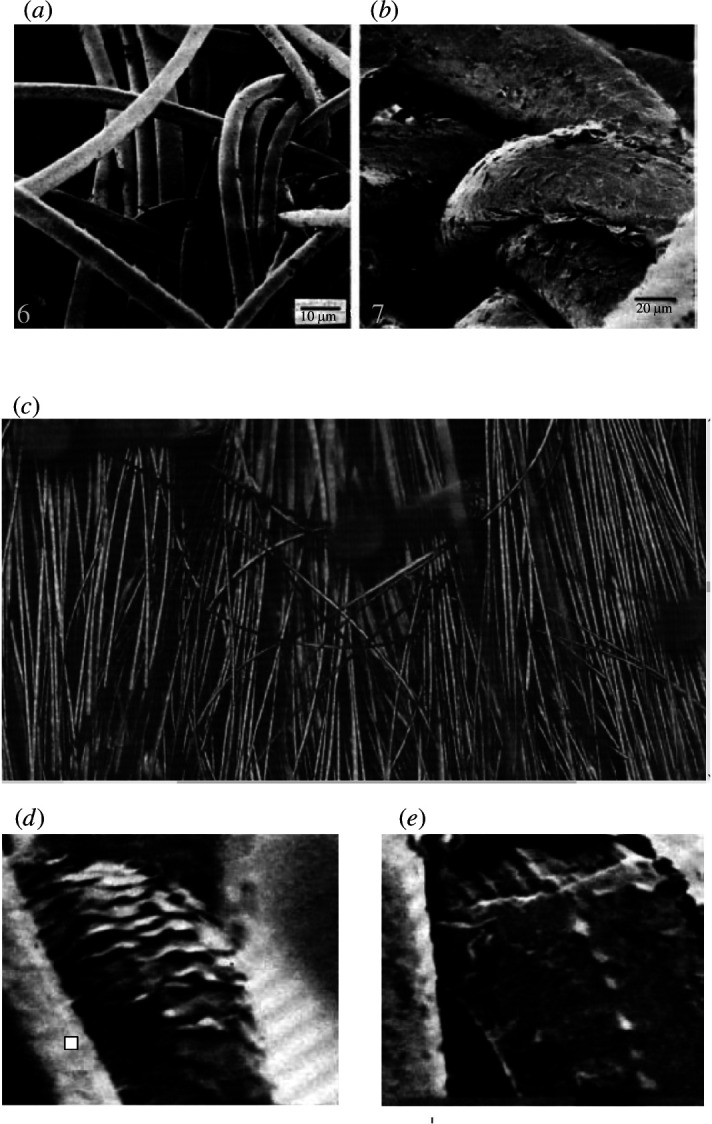
(*a*,*b*) SEM photos showing hair bending in tangles [25]. (*c*) Hairs looping over each other and over the teeth of a brush [33]. (*d*,*e*) Photos taken during a hair bending experiment in an SEM, showing: (*d*) cuticle lifting and (*e*) creation of a transverse crack [25].

Attention has focused on the combing/brushing of tangled hair. Hair forms tangles and knots very easily, more so if surface damage has occurred, increasing friction [32]. In a tangle, one hair bends over another with very high curvature, as shown in figure 5. This is possible owing to hair’s very high strain to failure. This curvature will create a complex pattern of stress, including high tensile and compressive stresses on the surface, and internal stresses including longitudinal shear. If one attempts to comb out a tangle, the tangle will move with the comb, causing the high local stresses to pass down the length of the hair until the tangle is resolved. Dynamic, impact loading may also occur. Since combing and brushing are repetitive actions, the loading is essentially cyclic, so fatigue failure is likely.

The above description constitutes the most convincing explanation for why hair fractures occur. It has been proposed and discussed by two research groups: Swift *et al.* (e.g. [25,31,34]) and Kamath *et al*. [10,35]. In the 1970s, Swift *et al*. conducted innovative experiments in which individual hairs and tangles were loaded inside a scanning electron microscope (SEM), allowing fracture events to be observed as they happened. Swift placed great emphasis on the shear stress which develops internally during bending, and which, in his view, is the primary cause of longitudinal crack propagation [31]. Robbins *et al*. conducted combing experiments, noting that hairs often impacted on each other. They defined different types of hair breakage (see above) and conducted controlled impact tests on individual hairs [10,33].

The contribution of these workers has been highly significant and well developed in many papers. A major limitation of this body of work, however, is the lack of quantification. There has been no significant attempt to predict the stresses and strains that will arise when hairs come into contact with a brush/comb or when they bend over each other in a tangle. No controlled experiments have been devised to simulate these actions and generate splitting in quantitative experiments. This has provided the main motivation for the present work.

## 2. Methods and materials

We used hair from two donors, both of whom are researchers and authors on this article. One author (R.E.-C., female, aged 45 years) had frequently experienced split ends: her hair is described here as ‘low quality’. The other author (R.T., male, aged 24 years) had not experienced split ends or any other problems with hair quality: his hair is described here as ‘control’. Both donors have straight hair with no curling: this was a deliberate choice because curly hair is known to have different structural features and different (in some cases inferior) mechanical properties, making it more complex to investigate [12].

**Figure 6.**
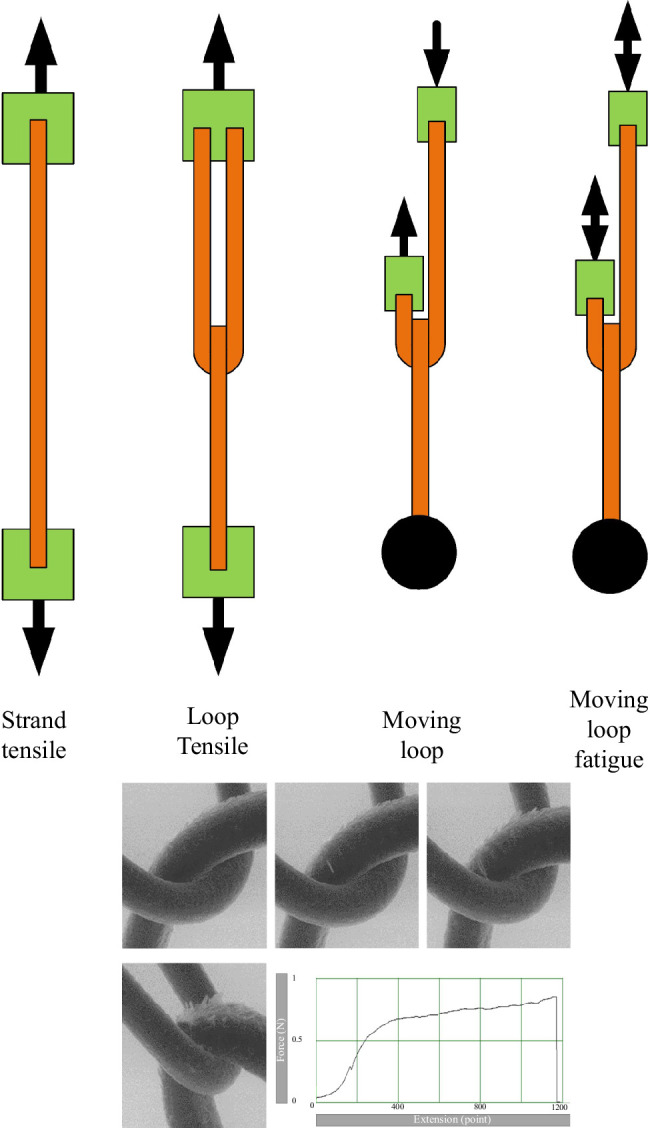
Schematic illustration of the four test types along with a series of photos and a typical force/displacement plot for the loop tensile test.

Hair strands were tested in one of four ways (see figure 6).

### 2.1. Strand tensile test

The test was carried out in a Zwick testing machine operating in tensile mode. The ends of the strand were glued to cardboard tags giving a test length of 40 mm. The tags were secured in the grips of the testing machine and the sample subjected to axial stretching at a rate of 20 mm min^−1^ until failure. Force was measured continuously.

### 2.2. Loop tensile test

This test was carried out in the same testing machine as the strand tensile test. Two strands were looped over each other. The ends of one strand were attached to the upper grip and those of the other strand to the lower grip, with test lengths of 40 mm. Tensile stretching was applied at 20 mm min^−1^ until failure, with continuous force measurement.

### 2.3. Moving loop test

A new testing machine was made for this test, consisting of two attachment grips connected to linear actuators, controlled by LabView software. Two hair strands of lengths of 100 mm were formed into a loop as for the loop tensile test. The two ends of the lower strand were attached to a weight. The ends of the upper strand were attached to two separate grips. These grips were then caused to move up and down with an amplitude of 20 mm. The movements were out of phase so that while one grip was moving down, the other was moving up. Thus, a length of 40 mm of the upper strand moved back and forth through the loop, while the lower strand remained in a fixed position. After the entire length of the strand had been moved through the loop, the test was complete, the test strand (upper strand) having either failed or survived.

### 2.4. Moving loop fatigue test

This is the same test as the moving loop test except that the movement of the grips was cycled back and forth with a frequency of 1 Hz, essentially repeating the moving loop test a number of times. The test was continued until failure occurred, recording the number of cycles to failure, defined as complete breakage of the test strand into two pieces.

The strand tensile test is the same type of test which has been carried out by previous workers and extensively reported in the literature. The loop tensile test was previously carried out by Swift *et al*. inside an SEM, but without measurement of force and displacement. The moving loop and moving loop fatigue tests have not been previously performed.

To investigate the effect of a common type of hair treatment, some control samples were subjected to bleaching using a commercial product (*L’Oréal Ultra-Lightening Excellence Pure Blonde 01*) which contains hydrogen peroxide and ammonium hydroxide. The product was applied to samples for 45 min, which is the manufacturer’s recommended time, and also for shorter and longer times (20 and 80 min) for comparison. Four samples were prepared in each case and tested using the moving loop fatigue test.

An optical microscope was used to measure the diameter of each hair strand and to determine the mode of fracture. Selected specimens were examined at higher magnification in an SEM. To investigate statistical significance between groups the *t*‐test was used, with a critical *p* value of 0.05.

## 3. Results

**Figure 7.**
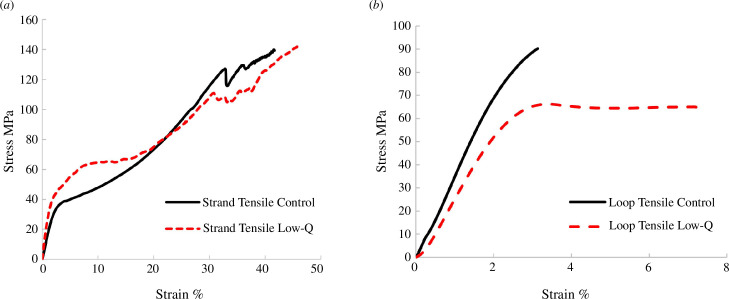
Results from: (*a*) strand tensile and (*b*) loop tensile tests.

Figure 7 shows stress/strain curves for the strand tensile and loop tensile tests. Stress is defined as force divided by the unloaded area of the cross-section of the hair strand for the strand tensile test. For the loop tensile tests, we define a nominal stress as the force divided by twice the cross-section area, this being the stress in the test strand at any point remote from the loop. This is a nominal stress because it does not take account of stress conditions in the loop itself. Diameter was measured at several points along each strand and averaged. Hair diameter varied considerably from 57 to 104 μm, but the average diameter was almost the same for the low-quality hair (80 μm) and the control hair (83 μm).

A minimum of five samples was tested in each group: figure 7 shows one typical curve for each, being the one whose tensile strength was closest to the average. However, these results were characterized by a lot of scatter. Table 1 records the yield strength (defined as stress at 5% strain) and the ultimate tensile strength (defined as the maximum stress achieved). Yield strength is not recorded for the loop tests because some samples failed before reaching 5% strain. Standard deviations are high, especially for the strand tensile tests. There was no significant difference between control and low-quality hair for any of the strength parameters, though tensile strength in the loop tests did approach significance (*p* = 0.061), with the low-quality hair being 31% weaker on average.

**Table 1.** Summary of results from the tensile tests (s.d. in parentheses).

test type	hair type	yield strength (MPa)	tensile strength (MPa)
strand tensile	control	47.1 (23.0)	153.0 (59.4)
strand tensile	low quality	57.3 (27.3)	144.2 (56.7)
loop tensile	control	n/a	95.2 (34.5)
loop tensile	low quality	n/a	65.4 (13.5)

In the strand tensile tests, the predominant failure mode was flat (figure 4*a*) with some small steps (figure 4*b*). Loop tensile tests showed mostly stepped fractures; in some cases, the steps were quite long (several hundred micrometres) and so may have started out as splits. Truly fibrous fractures (as in figure 4*c*) were not seen but, on close inspection in the SEM, some amount of fibrous separation near the fracture was common.

**Figure 8.**
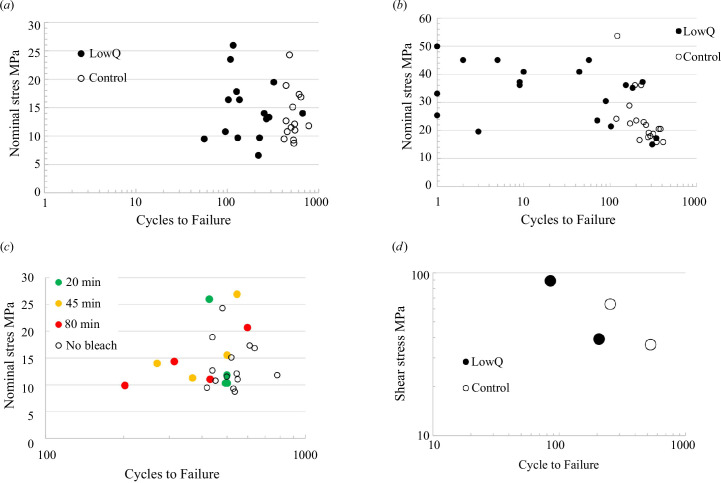
Results from the moving loop fatigue tests. (*a*) Low quality versus control at 14 g applied load; (*b*) low quality versus control at 26 g applied load; (*c*) bleached control samples (three different bleaching times) versus unbleached controls at 14 g load; (*d*) average values of cycles to failure and calculated average shear stress for control and low-quality samples at 14 and 26 g loads.

Figure 8 shows results from the moving loop tests, plotting the number of cycles to failure as a function of the nominal stress. Tests were carried out with two different values of the weight: 14 and 26 g. These values were chosen following preliminary trials which showed that they caused failures after a number of cycles in the range 1–1000. These weights represent approximately 10–30% of the force needed for failure in the loop tensile tests. If failure occurred during the first cycle, this constitutes a failure in the moving loop test. If failure did not occur, the test was continued as a moving loop fatigue test. Table 2 shows the average number of cycles to failure for each group.

**Table 2.** Average values of cycles to failure for each tested group, along with calculated shear stress and stress intensity for the non-bleached groups.

hair type	load (g)	average cycles to failure	average shear stress *τ* (MPa)	average stress intensity *K* (MPa√m)
low quality	14	207	39.2	0.44
low quality	26	85	89.1	1.00
control	14	531	36.2	0.41
control	26	255	64.2	0.73
bleached 20 min	14	480		
bleached 45 min	14	421		
bleached 80 min	14	386		

Regarding the tests with a weight of 14 g (figure 8*a*), there is a certain amount of scatter in the number of cycles to failure for a given stress, but this is quite typical of fatigue data for relatively brittle materials. There is a clear distinction between the control and low-quality samples, with the control samples lasting for more cycles, and this is statistically significant (*p* < 0.001). A similar pattern emerges for the tests conducted with a weight of 26 g (figure 8*b*) except that here, there is much more scatter in the low-quality samples, with several failing in the first cycle and approximately half failing in 10 cycles or less.

**Figure 9.**
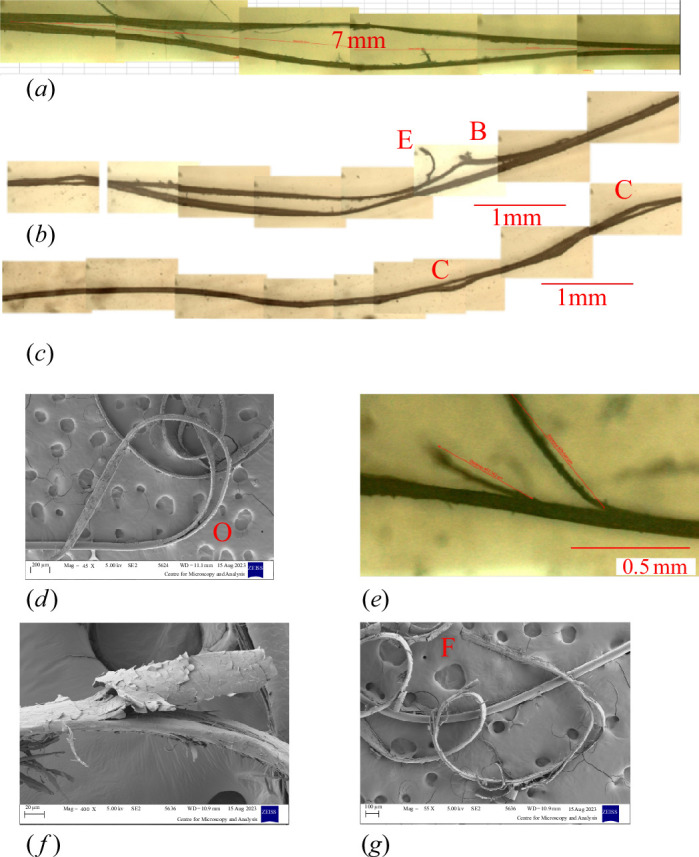
Examples of the two splitting modes observed in moving loop fatigue tests, in low-quality (*a–d*) and control (*e–g*) samples. (*a*) A centre split which has propagated to a length of 7 mm. (*b*) Another long split which has already broken on one side (marked ‘B’). There is also a small edge split ‘E’. (*c*) Two short centre splits ‘C’. (*d*) SEM image showing one end of a centre split ‘O’. Note how the two halves of the split curl around in a ribbon shape. (*e*) Two short edge splits. (*f*) SEM image showing lifting of cuticle tiles and fracture in the cortex for a short edge split. (*g*) Several edge splits, one of which has propagated to break the strand at ‘F’.

The predominant failure mode for moving loop fatigue tests was splitting, but two distinct types of splitting were found, as illustrated in figure 9. The low-quality hair typically failed from splits that initiated inside the hair strand, close to the centre. These splits usually propagated for a considerable distance—more than 5 mm and sometimes for the complete test length of 40 mm—before the strand finally broke into two pieces. The broken halves tended to curl up and this, along with their reduced thickness, gave them a ribbon like appearance (figure 9*d*). This occurred in all tests carried out with a load of 14 g and in the majority of 26 g tests.

By contrast, the splits in the control hair almost always initiated from the strand’s surface, sometimes appearing first as lifted cuticle tiles and gradually propagating inwards at a shallow angle (figure 9*e,f*). Noting the morphology of the cuticle tiles, we can deduce that, in most cases, splits propagated up the hair, i.e. towards the hair root. This was true for both 14 and 26 g loads. Typically, many such splits developed with lengths less than 1 mm (figure 9*g*) before one propagated sufficiently to cause breakage. We refer to these two splitting modes as ‘centre splitting’ and ‘edge splitting’. In some cases, both modes were present when the sample was examined after breaking so it was difficult to be sure which predominated. So, some tests were carried out in which the cycling was interrupted periodically for microscopic examination. In these tests, centre splitting was observed (and was the cause of failure) in almost all the low-quality samples (10 out of 11: in one sample, no splits were seen before failure); figure 9*a–c* are examples. In some cases, edge splits also formed (figure 9*c*) but did not cause failure. All control samples subjected to interrupted testing (6/6) failed by edge splitting, an edge split finally propagating through to the far side of the strand. A centre split was seen in one sample but it was short (<1 mm) and did not cause the failure.

Regarding the bleached samples (figure 8*c*), there was no significant effect on the number of cycles to failure after a 20 min treatment (*p* = 0.32) but after 45 and 80 min, there were significant reductions compared with unbleached controls (*p* = 0.024 and 0.039, respectively). On the plot, it is clear that this was owing to some (but not all) of the samples failing in fewer cycles than the unbleached controls. Regarding the mechanism of failure, there was a tendency for more central splitting in the samples bleached for longer times, though more testing would be needed to confirm this trend.

## 4. Discussion

The new experiments presented here have shown considerable success in being able to distinguish between hair from two different donors, one of whom experienced split ends while the other did not. The loop tensile test proved superior to the more commonly used strand tensile test, showing less scatter and a larger average difference between hair types, which just missed significance. However, the moving loop fatigue test proved to be by far the best, generating the splitting mode and showing clear differences both in number of cycles to failure and in the type of splitting which occurred. This test was also able to detect the effects of bleaching.

Some insight into the mechanics of the two splitting modes can be achieved using the following simple calculations. Central splitting involves the initiation and growth of a crack oriented longitudinally, along the hair shaft. The hair in the loop is being loaded in bending, so the stress available to cause this longitudinal crack is the shear stress in the longitudinal plane (the horizontal plane in the orientation of this test). Assuming, for simplicity, that the loading is three point bending, with a pair of forces *F*/2 applied through the two strands and a balancing force *F* acting through the centre of the loop, then the largest shear stress occurs on the mid-plane and can be expressed in terms of the applied load and the hair radius *r*, as


(4.1) $$\tau =\frac{4F}{3\pi r^{2}}.$$

We assume that the hair strand has a constant radius, a reasonable assumption for straight hair, ignoring the more complex structure of curly hair. This stress is simply related to the nominal stress defined above, being larger by the factor 8/3. The assumption of three point bending here is certainly a simplification owing to the degree of contact between the two strands, but if we take the other extreme assumption (perfect contact), then the equation is the same but with the factor of 4/3 being replaced by unity. Figure 8*d* shows results from the moving loop tests (at both 14 and 26 g loads), plotting the average shear stress and the average cycles to failure for each hair type and each applied load. These values are given in table 2.

A longitudinal crack which forms in a loop tensile test will not be able to extend very far because to do so, it would travel out of the loop, into parts of the hair strand in which there would be no stress to drive it. We assume that these cracks will extend to a total length (tip-to-tip) approximately equal to the diameter of the strand. In the moving loop test, on the other hand, this microcrack may be able to propagate by growing as the loop moves. According to fracture mechanics theory, a crack of length *a* will propagate under an applied stress *σ* if the stress intensity *K* is greater than the fracture toughness *K*_c_. The stress intensity in this case is given by


(4.2) $$K=Q\tau \sqrt{\pi a}.$$

Here, *a* is the half-length of the crack (thus, equal to *r* in this case) and *Q* is a factor which depends on the shape and size of the crack and the type of loading, in this case a reasonable estimate for *Q* being 1.0 [36].

Table 2 shows values of *K* obtained for each group using the average stress (as in figure 9) and average strand radius.

At 14 g load, *τ* is similar for the two hair types, as is *K*, but the low-quality hair failed by centre splitting while the control hair failed by edge splitting. This suggests that centrally located longitudinal cracks do not form at a shear stress of 36 MPa in the control hair or, if they do, they fail to propagate at a *K* of 0.41 MPa√m. For the low-quality hair, on the other hand, we are clearly above the limits for both initiation and propagation at 39.2 MPa and 0.44 MPa√m. However, this initiation and propagation of cracks does not occur immediately but rather requires some cycles. At 26 g, we are still very much in the fatigue regime for the control hair, but about half of the low-quality hair failed immediately (or in very few cycles) suggesting that 89 MPa and 1.0 MPa√m are approaching the static shear strength and the fracture toughness for longitudinal cracks in this material.

These values of *τ* and *K* are surprisingly high, considering the measured tensile strength of the low-quality hair (144.2 MPa) and the toughness estimate by Kamath and Weigmann mentioned above which (when converted into a *K* value) give *K*_c_ = 1.1 MPa√m. One might have expected a greater degree of anisotropy in both strength and toughness, considering the results mentioned above for other fibrous keratin-based materials. Two factors might be relevant here. First, the longitudinal crack in the loop is subject to a compressive force which will tend to press the two crack surfaces together and help it to resist propagating under the applied shear, as a result of friction between the crack faces. Second, hair is known to be strain-rate sensitive, displaying greater strength and stiffness when loaded quickly [6]. There will be a difference of several orders of magnitude between the strain rate applied during moving loop tests and that applied during the static tensile tests.

Edge splitting is a more complex phenomenon as regards its mechanics. As the hair strand passes through the loop, it will experience very high tensile loading on part of its surface. Assuming it bends tightly against the other hair strand then, if the two strands have the same diameter, a tensile strain of 50% will occur, which is similar to the failure strain seen in tensile tests (figure 7). Small cracks are likely to form at different points along the strand. A crack of this type experiences tensile stress but very little shear stress: in theory, the shear stress is zero at the surface and rises in a parabolic manner towards the centre. However, if the crack turns towards the longitudinal direction the tensile stress will cause shear-type deformations of the crack faces, creating stress intensity (*K*) values in Mode II and Mode III (in-plane and out-of-plane shear, respectively). It would be these stress intensities which would drive crack propagation at a shallow angle, in a direction close to the longitudinal one where the material is weakest owing to anisotropy. We observed that most of these splits began with the lifting of cuticle tiles, which are layered in a particular direction; as a result, the majority of splits propagated up the hair (towards the root). This would be more likely to occur when the strand moved over the stationary loop in that direction, which is opposite to the normal direction of brushing, which would of course be towards the tip of the hair. In this respect, our moving loop test, in which the hair is moved in both directions, differs from normal brushing action in a way that may encourage splits to form more easily than they would normally do.

Thus, it can be seen that centre splitting is entirely controlled by shear stress while edge splitting requires a mixture of tensile and shear stress, with the tension driving initiation and early growth and shear taking over as the split extends. Quantifying these stresses and stress intensities in a theoretical model is difficult because one needs to take account of the highly nonlinear stress–strain behaviour of the material. Such a model should also include the other effects mentioned above: transverse compression on the longitudinal crack, and the effect of strain rate.

These various results, taken together, suggest that the principal difference between the control and low-quality hair lies in the anisotropy of the material. Tensile strength (for stressing in the longitudinal direction) is similar in both types, but under moving loop testing the low-quality hair fails sooner by the creation of central splits owing to its poor transverse strength leaving it susceptible to longitudinal shear stress. Normal, control hair has sufficient transverse strength to resist this failure mode, eventually failing (after more cycles) owing to surface-initiated cracks (or small pre-existing surface flaws) caused by very high, but very localized, cyclic tension. This interpretation is consistent with the known effects of bleaching and other treatments [8,26] which tend to affect the bonds between the fibrous cortical cells (figure 1), selectively reducing their ability to resist transverse tension and longitudinal shear.

This work had several limitations. We used only one rate of displacement in the static tests and one frequency in the moving loop tests and thus, we did not investigate the effects of strain rate, which are known to be significant in keratin. Humidity and temperature, factors which can affect hair strength, were not controlled. Combing of tangled hair likely involves a degree of impact loading which we did not replicate. Samples were used from only two different individuals, both having straight hair.

This work constitutes a first step in the development of a quantitative approach to the biomechanics of hair splitting. A test has been developed which is able to distinguish between hair which is susceptible to splitting and hair which is not, generating different failure modes and different numbers of cycles to failure. The same test was also able to demonstrate the deterioration of hair quality caused by bleaching. Different splitting modes have been identified for the first time, emphasizing the complex nature of this failure process. These results provide support for the hypothesis of Swift, that splitting occurs owing to the high longitudinal shear stresses that arise in tangles, but further work is needed to investigate this and other potential causes. This work paves the way for future studies, including a more comprehensive experimental programme involving a large number of donors with different hair types (including curly hair), study of the effects of humidity, temperature and different treatments and the development of a theoretical model to describe the biomechanics and fracture of the hair strand. Hair is a complex fibre composite material exhibiting structure at various length scales, as mentioned above. This aspect should be considered in future studies with respect to the splitting modes demonstrated here.

## Data Availability

All original data are available in the supplementary material [37].
